# Characterization data of reference materials used for phase II of the priority program DFG SPP 2005 “Opus Fluidum Futurum – Rheology of reactive, multiscale, multiphase construction materials”

**DOI:** 10.1016/j.dib.2023.108902

**Published:** 2023-01-13

**Authors:** U. Pott, C. Crasselt, N. Fobbe, M. Haist, M. Heinemann, S. Hellmann, D. Ivanov, C. Jakob, D. Jansen, L. Lei, R. Li, J. Link, D. Lowke, V. Mechtcherine, J. Neubauer, D. Nicia, J. Plank, S. Reißig, T. Schäfer, C. Schilde, W. Schmidt, C. Schröfl, T. Sowoidnich, B. Strybny, N. Ukrainczyk, J. Wolf, P. Xiao, D. Stephan

**Affiliations:** a Department of Civil Engineering, Technische Universität Berlin, Berlin 13355, Germany; b Bundesanstalt für Materialforschung und -prüfung, Berlin 12205, Germany; c GeoZentrum Nordbayern, Mineralogy, Friedrich-Alexander Universität Erlangen-Nürnberg, Erlangen 91054, Germany; d Institute of Building Materials Science, Leibniz Universität Hannover, Hannover 30167, Germany; e F. A. Finger-Institute for Building Material Science, Bauhaus-Universität Weimar, Weimar 99423, Germany; f Institute of Geosciences, Applied Geology, Friedrich-Schiller-Universität Jena, Jena 07749, Germany; g Institute for Particle Technology (iPAT), Technische Universität Braunschweig, Braunschweig 38106, Germany; h Department of Chemistry, Technische Universität München, Garching 85748, Germany; i Institute of Building Materials, Concrete Construction and Fire Safety (iBMB), Technische Universität Braunschweig, Braunschweig 38106, Germany; j Institute of Construction Materials, Technische Universität Dresden, Dresden 01062, Germany; k Construction and Building Materials, Technische Universität Darmstadt, Darmstadt 64287, Germany

**Keywords:** Portland cement, Limestone powder, Calcined clay, Sustainable cement, DFG SPP 2005

## Abstract

A thorough characterization of base materials is the prerequisite for further research. In this paper, the characterization data of the reference materials (CEM I 42.5 R, limestone powder, calcined clay and a mixture of these three components) used in the second funding phase of the priority program 2005 of the German Research Foundation (DFG SPP 2005) are presented under the aspects of chemical and mineralogical composition as well as physical and chemical properties. The data were collected based on tests performed by up to eleven research groups involved in this cooperative program.

**Specifications Table**

**Table:** 

Subject	Ceramics and Composites
Specific subject area	Building materials; Cement, limestone powder, calcined clay, LCC cement 70:30
Type of data	Table; Image; Figure
How the data were acquired	XRD; SEM; EN 196–1: 2016; EN 196–2: 2013; EN 196–3: 2017; EN 196–6: 2019; EN 196–11: 2019; EN 1097–7: 2008; ISO 13,320: 2020; ISO 9277: 2014
Data format	Raw; Analyzed
Description of data collection	All research institutions have received material from the same batch. A uniform test procedure was discussed, and the results of these tests were collected and evaluated.
Data source location	Ten universities and one research institute as shown in Tab.1 performed tests. The results were collected at TU Berlin, Germany.
Data accessibility	https://depositonce.tu-berlin.de/items/346e2a59–7eaf-413c-9796–3e5c8917caaf, doi:10.14279/depositonce-16,384.2[1]

## Value of the Data

• The aim was to characterize the raw materials as the basis for further research in the DFG SPP 2005 priority program.
• The extensive data set illustrates differences in reproducibility depending on the material and the method.
• Particularly large variations occurred in the particle size distribution of the limestone powder.
• The data can be used as benchmark values for other researchers and may be beneficial for researchers trying to optimize a method used in this study.
• The data can be reused by researchers who used the same material or method and need comparative values for their measurements.

## 1 Objective

Within the framework of the DFG SPP 2005 priority program a second funding phase containing eleven projects started in spring 2021. Two papers have already been published in Data in Brief for the first funding phase. For the second funding period, the projects have received new materials as a basis for their research. The aim of this dataset is to characterize the raw materials as the basis for further research in the priority program. The data presented will be cited by the imminent research articles by members of DFG SPP 2005.

## 2 Data Description

Table 1. lists the universities and the research institute involved in the characterization of the different raw materials.

**Table 1:** Universities and the research institute involved in the characterization.

No.	Affiliation
1	Bundesanstalt für Materialforschung und -prüfung
2	Bauhaus-Universität Weimar
3	Friedrich-Alexander-Universität Erlangen-Nürnberg
4	Friedrich-Schiller-Universität Jena
5	Leibniz Universität Hannover
6	Technische Universität Berlin
7	Technische Universität Braunschweig - iBMB
8	Technische Universität Braunschweig - iPAT
9	Technische Universität Darmstadt
10	Technische Universität Dresden
11	Technische Universität München

The majority of data are presented as boxplot diagrams. These diagrams include the median line (50th percentile), the range between 25th and 75th percentiles indicated as a box, the range within 1.5 times of the interquartile range (IQR) indicated as whiskers and outliers if present. For a specific explanation, the reader is referred to [2]. Moreover, the mean value is calculated based on the whole dataset (including outliers) and added to the boxplot as a hollow square. Each institute has been assigned a symbol for the entire paper to display the individual measured values. The exact assignment is anonymous. Nevertheless, this representation allows an evaluation of the reliability of the measured values. Based on this representation it can be determined, for example, that outliers always stem from different participants. The symbols of the individual measurements are shown on the right side of the boxplot. The curved line next to the individual measurements represents the measured values as a normal function (Gaussian distribution).

Four different materials were examined. A Portland cement CEM I 42.5 R, a limestone powder, a calcined clay, and a mixture of these three components plus additional anhydrite. The mixture is designated as LCC cement 70:30 (abbreviation LCC 70:30). It is composed as follows:

■ 51.87 wt.% CEM I 42.5 R
■ 15.61 wt.% Limestone powder
■ 2.52 wt.% Anhydrite
■ 30.00 wt.% Calcined clay

The name of the mixture reflects the initial letters of the main components and the ratio of the calcined clay (30 wt.-%) to the rest of the materials (70 wt.%). The properties of this mixture are of interest because it was developed along the lines of the so-called LC^3^ cements, developed at the institute EPFL STI IMX LMC [3,4]. Compared to pure Portland cement, the mixture is more sustainable due to the replacement of Portland cement with calcined clay and limestone powder.

### 2.1 Characterization Data of Oxide Composition and Phase Contents

**Fig. 1:**
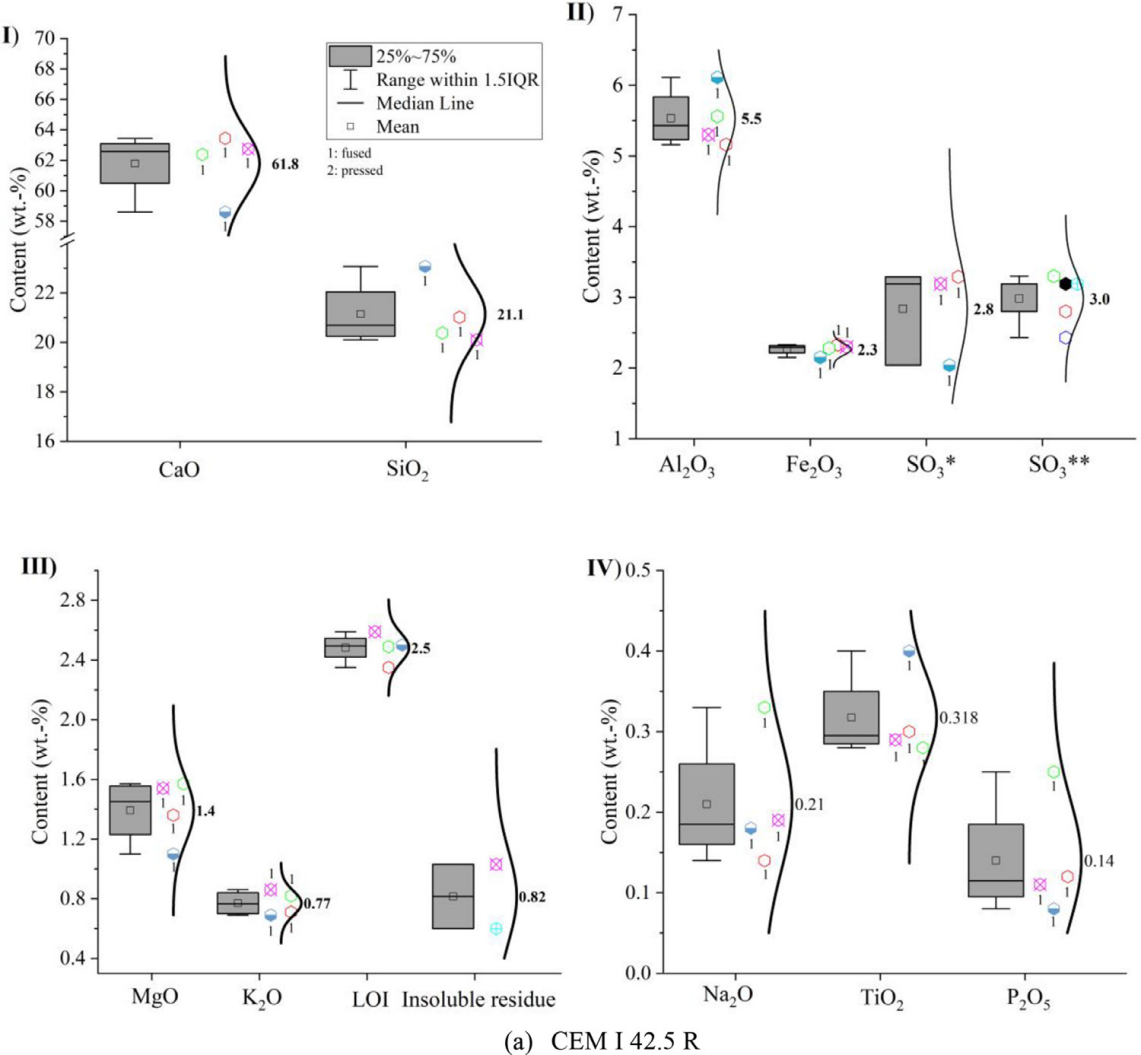

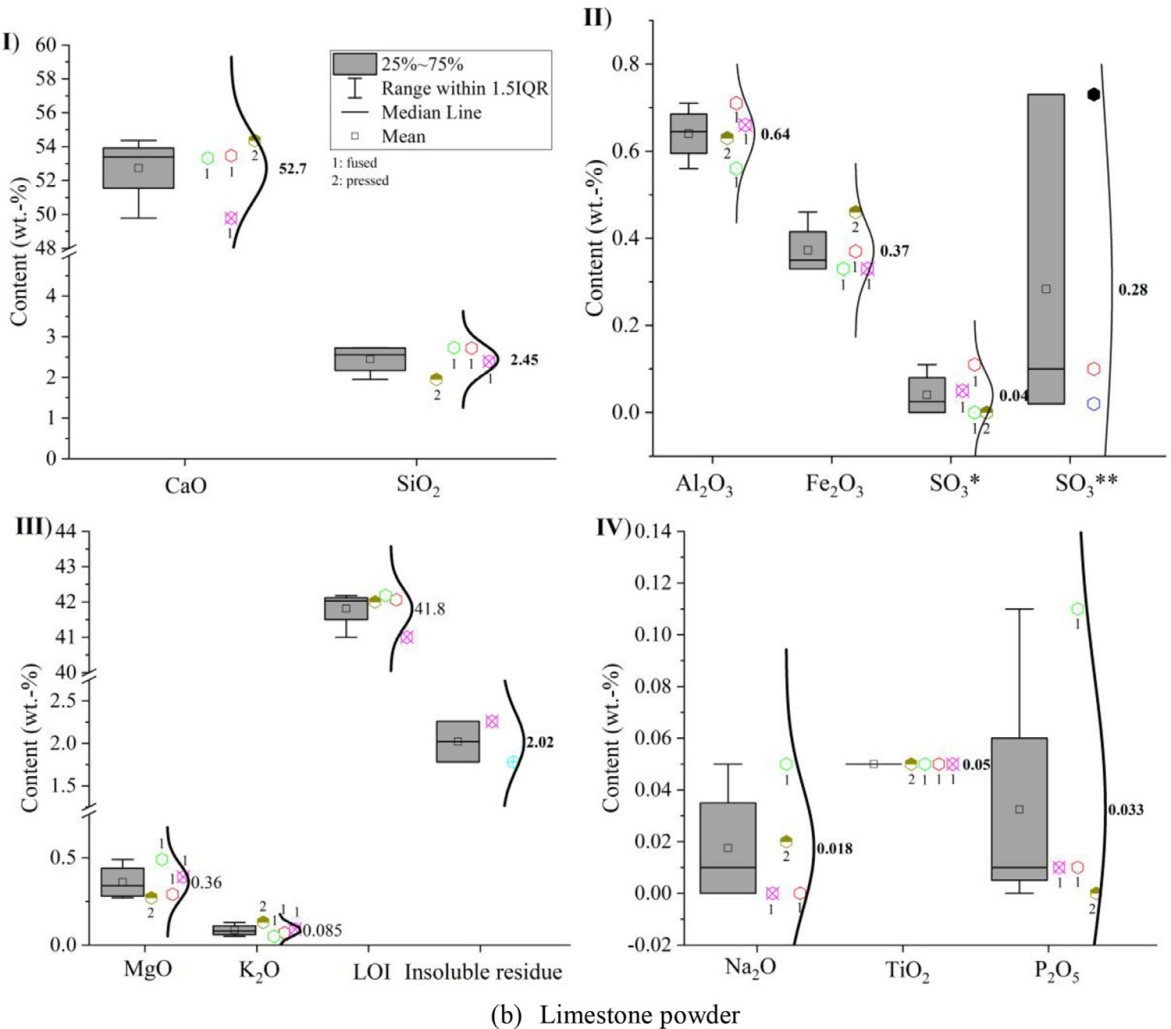

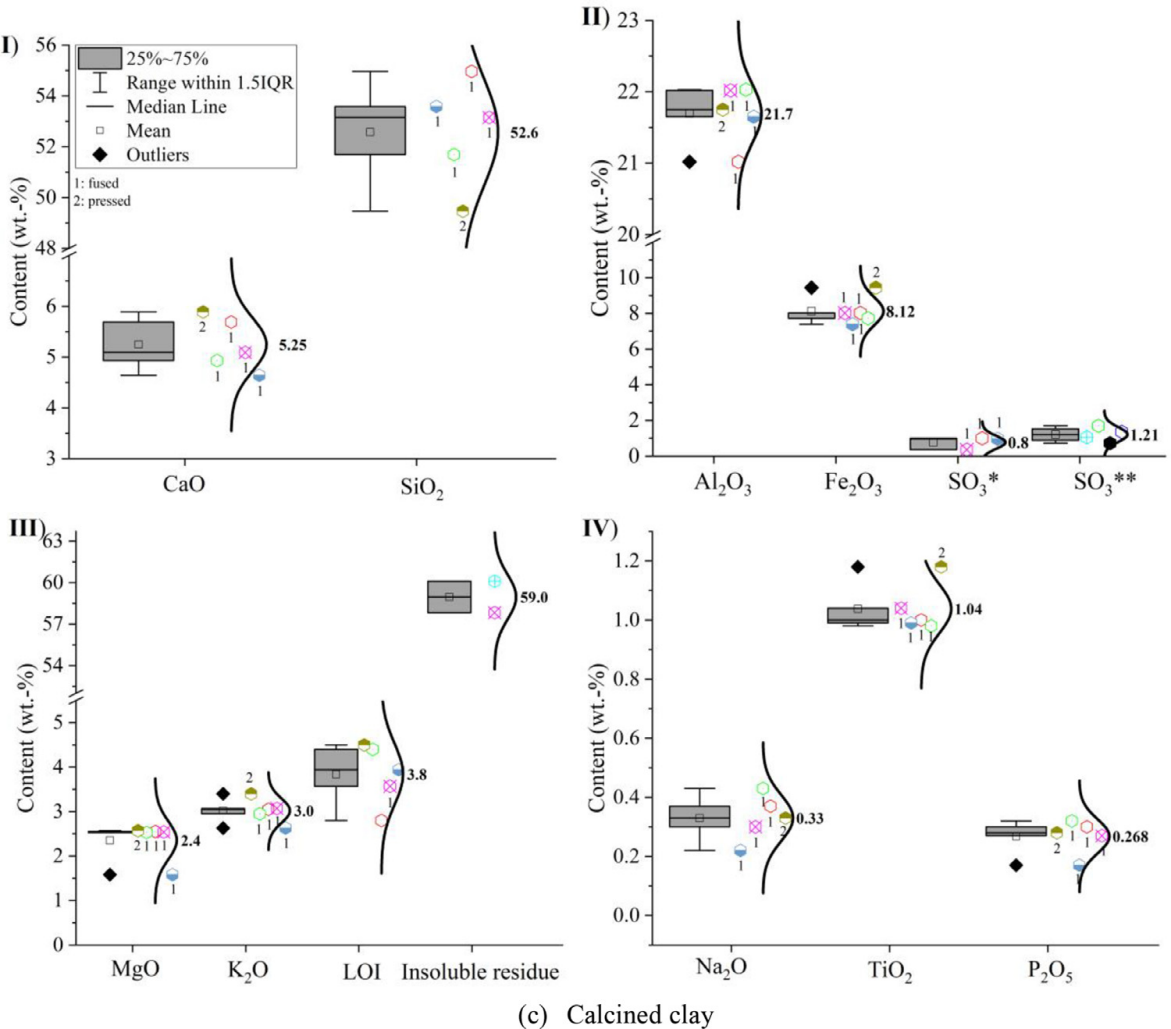

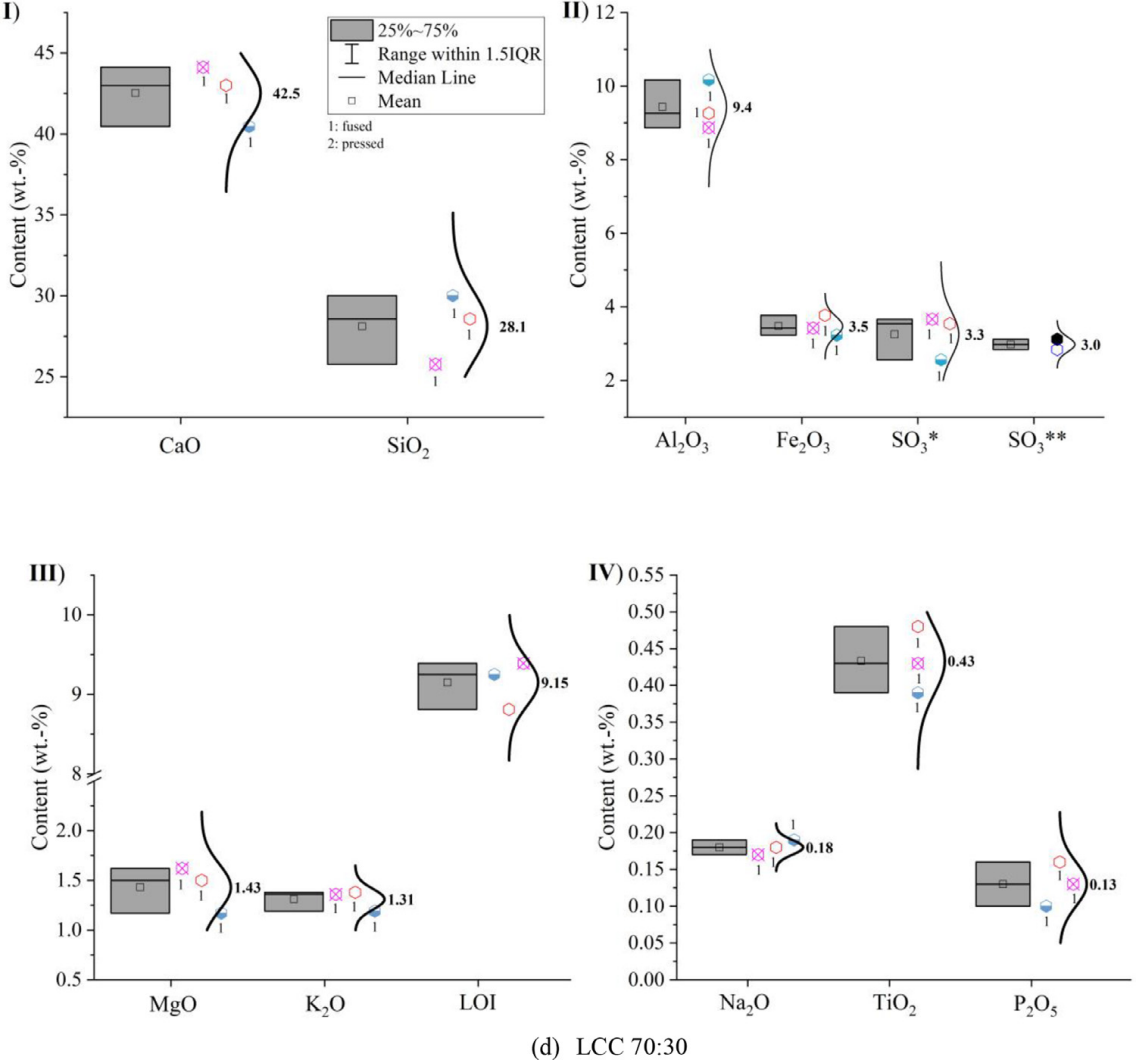
Oxide compositions of (a) CEM I 42.5 R, (b) limestone powder, (c) calcined clay and (d) LCC 70:30 measured by X-ray fluorescence analysis; (I) CaO and SiO_2_; (II) Al_2_O_3_, Fe_2_O_3_ and SO_3_; (III) MgO, K_2_O, loss on ignition (LOI) and insoluble residue; (IV) Na_2_O, TiO_2_ and P_2_O_5_.

Oxide composition, insoluble residue and loss on ignition (LOI) of CEM I 42.5 R, limestone powder, calcined clay and LCC 70:30 were measured according to EN 196-2: 2013 [5] and the results are shown in Fig. 1. In the sub-images (II) SO_3_* indicates that the value was obtained by X-ray fluorescence analysis (XRF) and SO_3_** indicates that the value was captured by conventional wet chemistry method. The numbers next to the symbols indicate whether a fused (1) or pressed (2) tablet was measured.

**Fig. 2:**
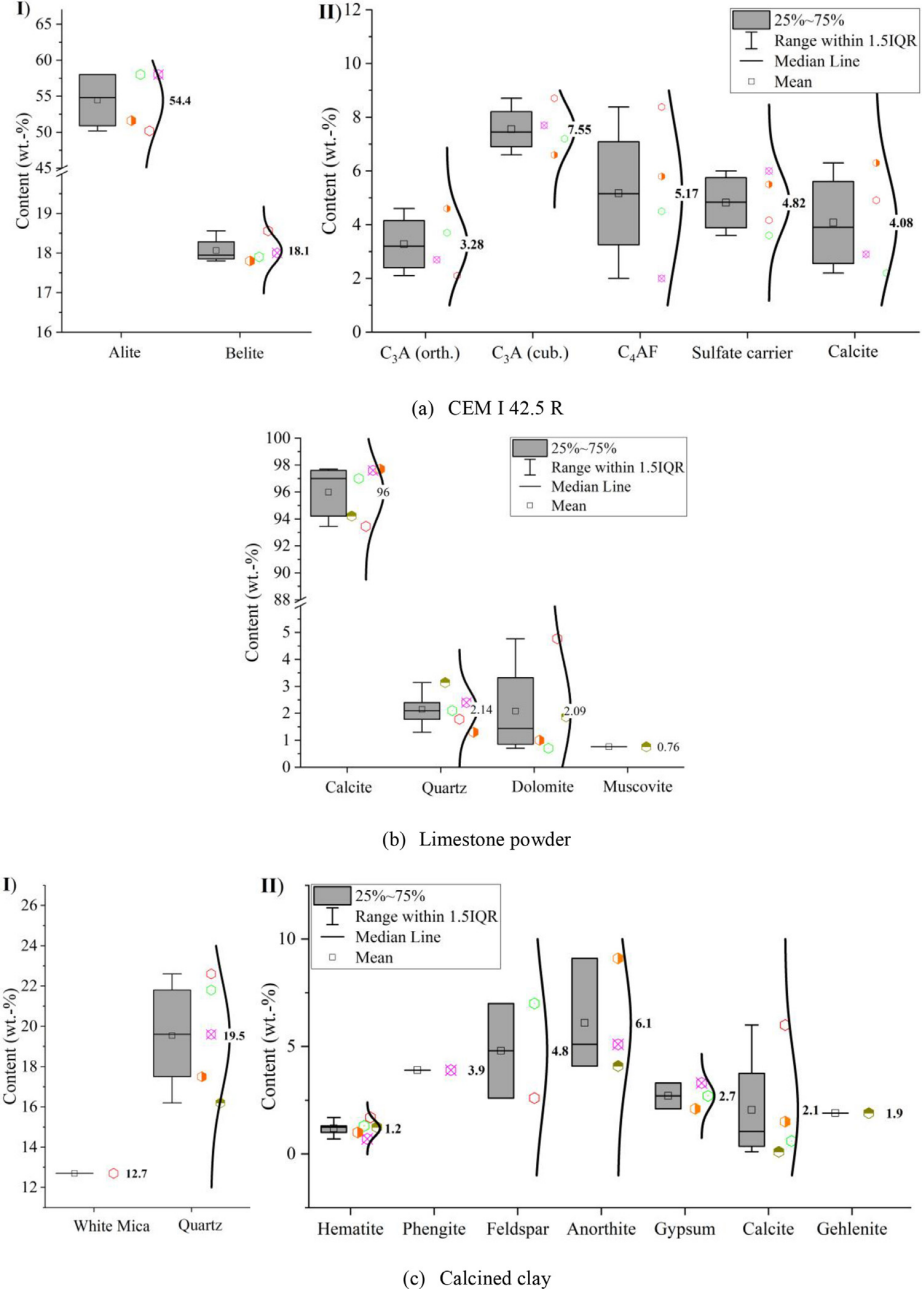
Phase contents determined by powder-X-ray diffraction (p-XRD) of (a) CEM I 42.5 R (I: C_3_S, C_2_S; II: C_3_A (orth.), C_3_A (cub.), C_4_AF, sulfate carrier and calcite), (b) limestone powder (calcite, quartz, dolomite, muscovite) and (c) calcined clay (I: white mica, quartz; II: hematite, phengite, feldspar, anorthite, gypsum, calcite, gehlenite).

Fig. 2 shows the phase contents of CEM I 42.5 R, limestone powder and calcined clay determined by powder-X-ray diffraction (p-XRD) in combination with the Rietveld quantification method [6].

### 2.2 Characterization Data of Physical Properties

**Fig. 3:**
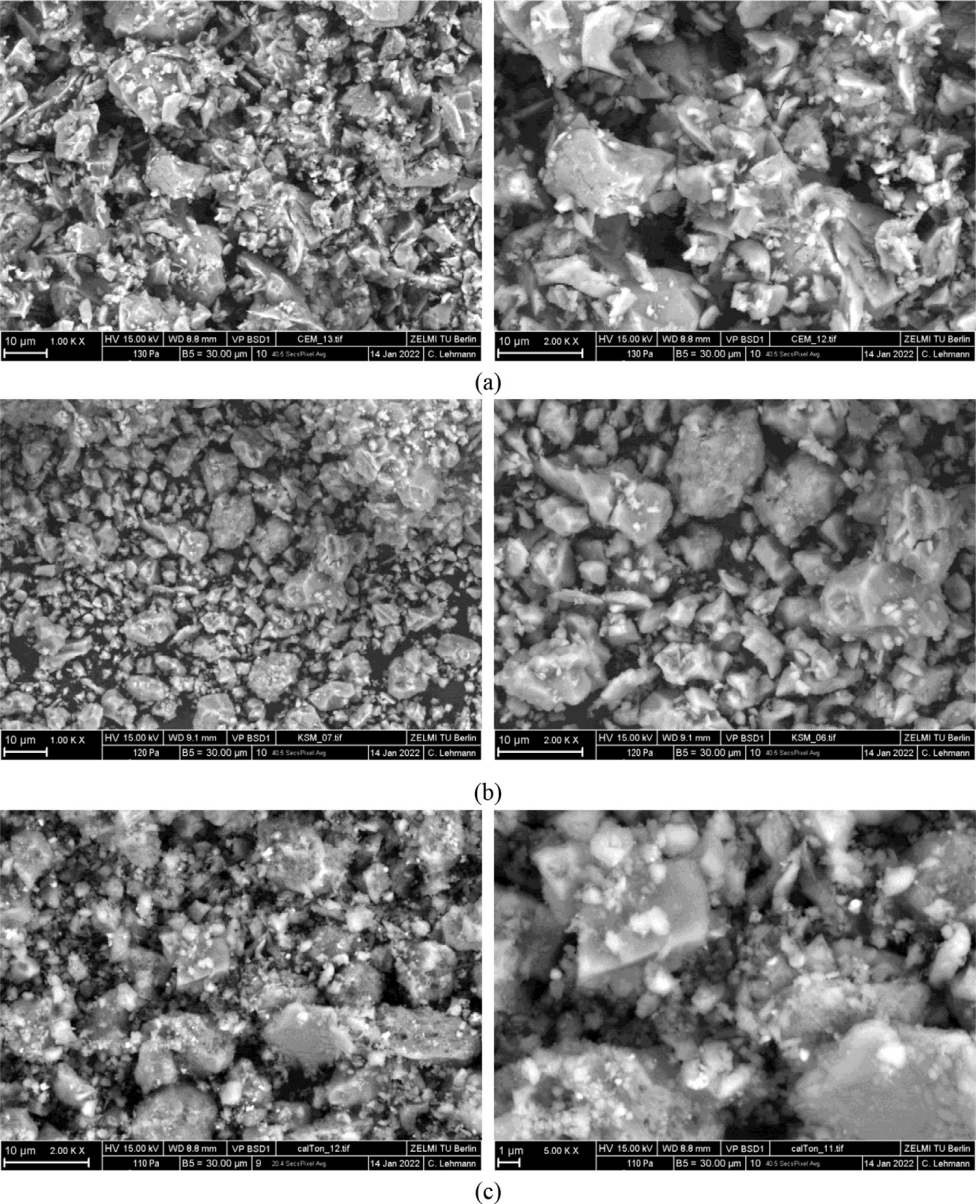
SEM pictures of (a) CEM I 42.5 R (b) limestone powder and (c) calcined clay at different magnifications.

Fig. 3 shows selected SEM pictures of CEM I 42.5 R, limestone powder and calcined clay at different magnifications.

**Fig. 4:**
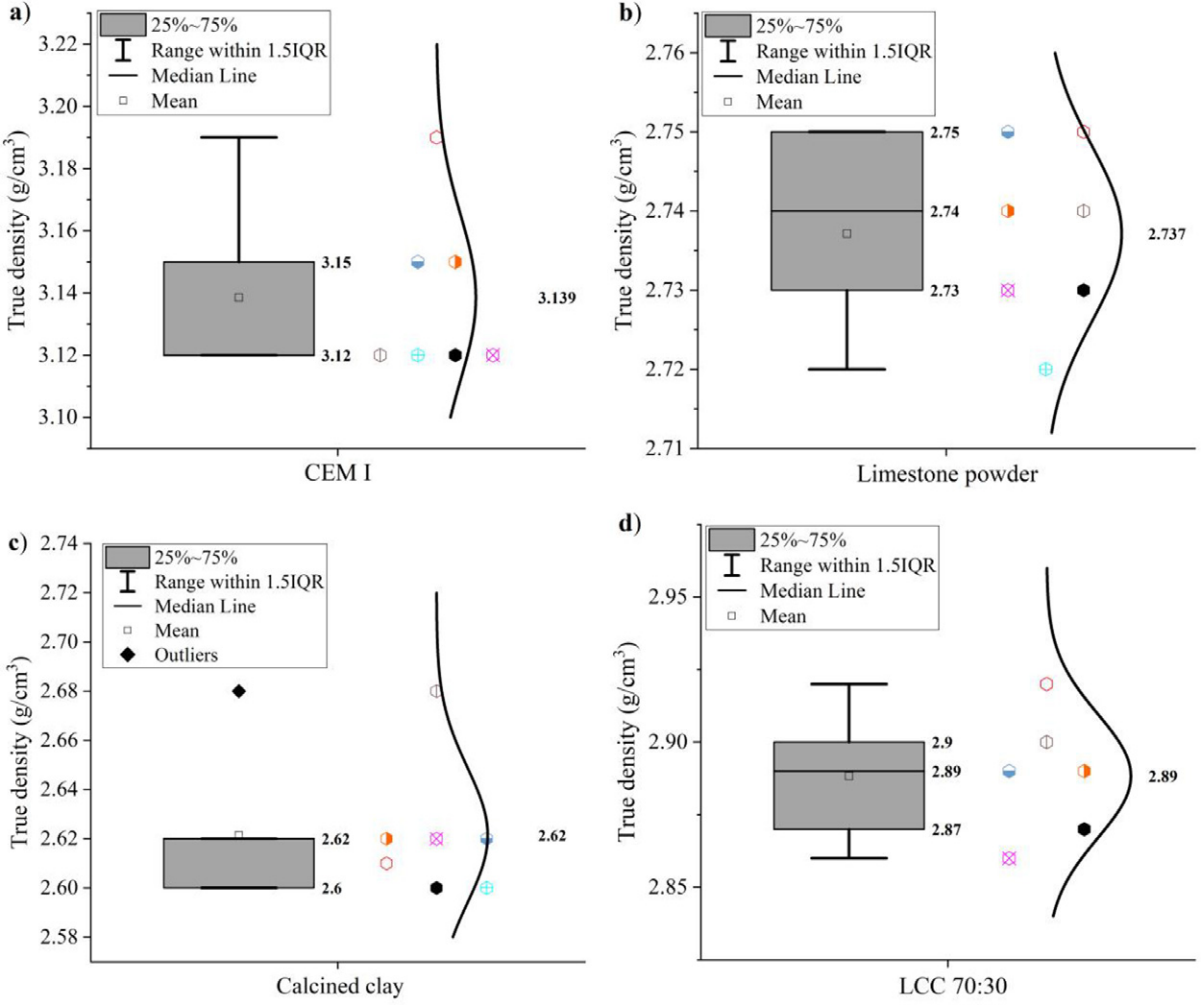
True densities of (a) CEM I 42.5 R, (b) limestone powder, (c) calcined clay and (d) LCC 70:30.

The true densities of CEM I 42.5 R, limestone powder, calcined clay and LCC 70:30 were measured by the Helium pycnometer method according to EN 1097–7: 2008 [7]. Results are shown in Fig. 4.

**Fig. 5:**
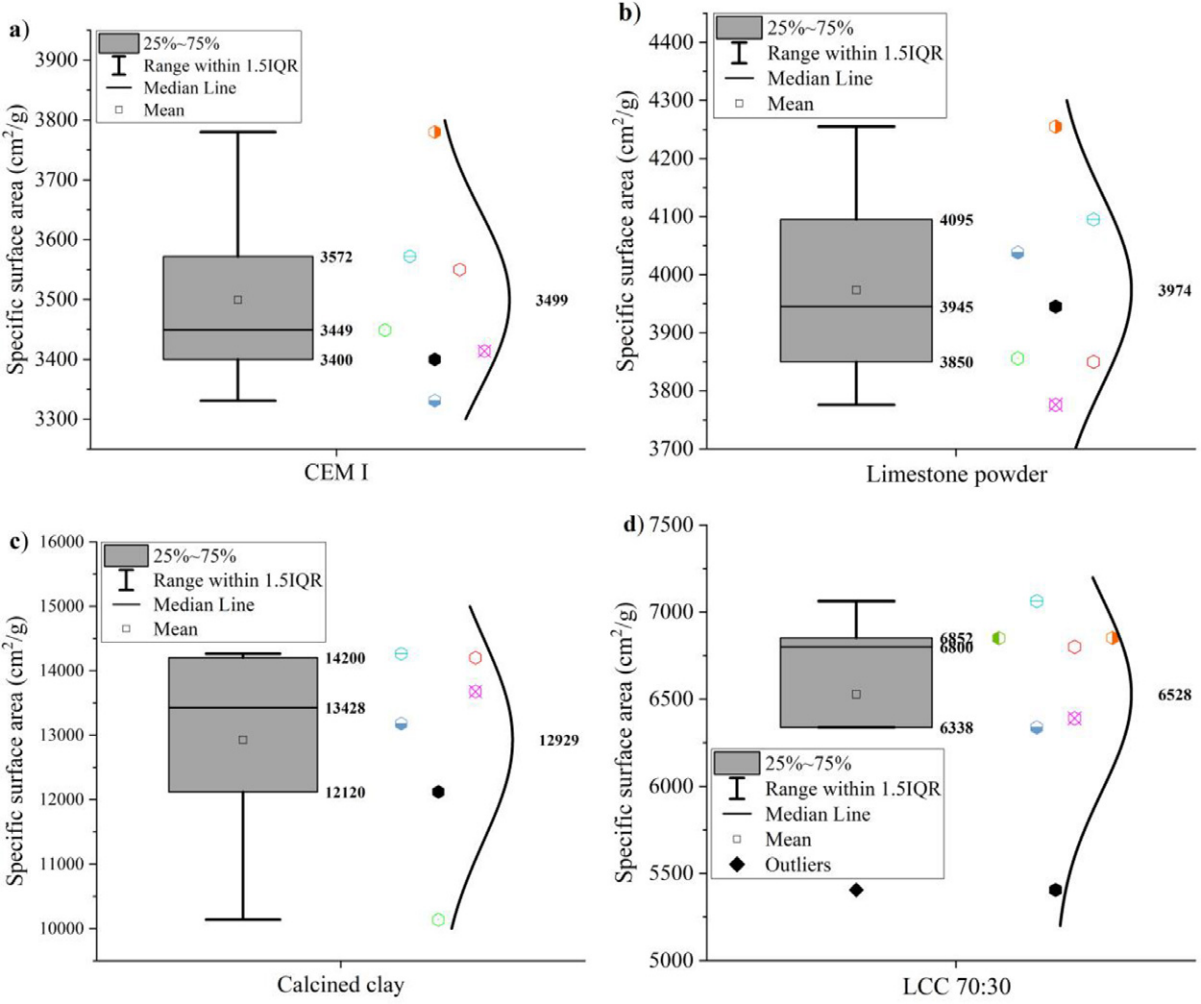
Specific surface area of (a) CEM I 42.5 R, (b) limestone, (c) calcined clay, and (d) LCC 70:30 measured by the Blaine method.

The specific surface areas of CEM I 42.5 R, limestone powder, calcined clay and LCC 70:30 were measured by the Blaine method according to EN 196–6: 2019 [8] The results are shown in Fig. 5.

**Fig. 6:**
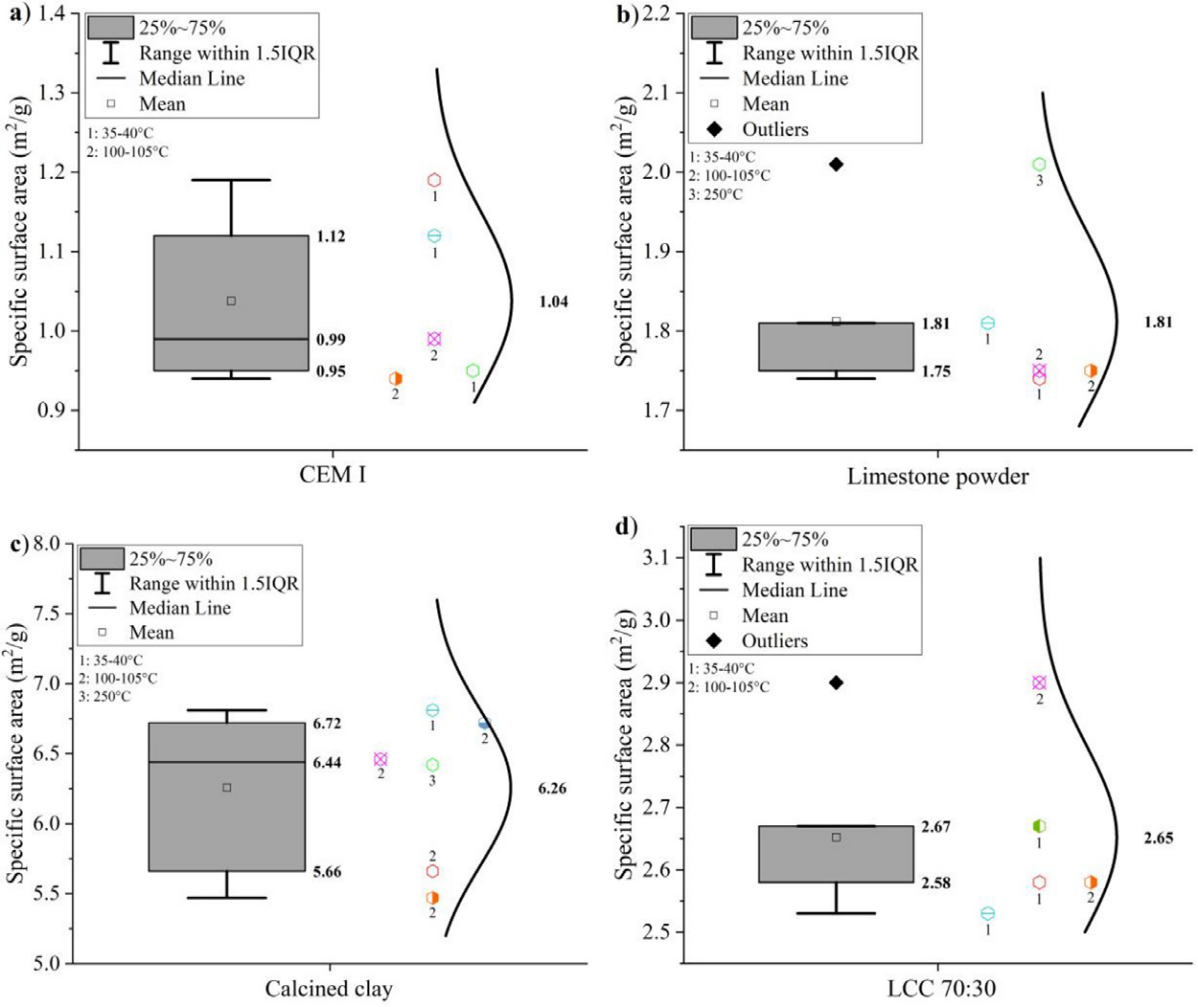
Specific surface areas of (a) CEM I 42.5 R, (b) limestone powder, (c) calcined clay and (d) LCC 70:30 measured by the BET method.

The specific surface areas of CEM I 42.5 R, limestone powder, calcined clay and LCC 70:30 were measured by the BET method according to ISO 9277: 2014 [9]. Results are shown in Fig. 6. The numbers next to the symbols indicate the temperature for sample preparation.

**Fig. 7:**
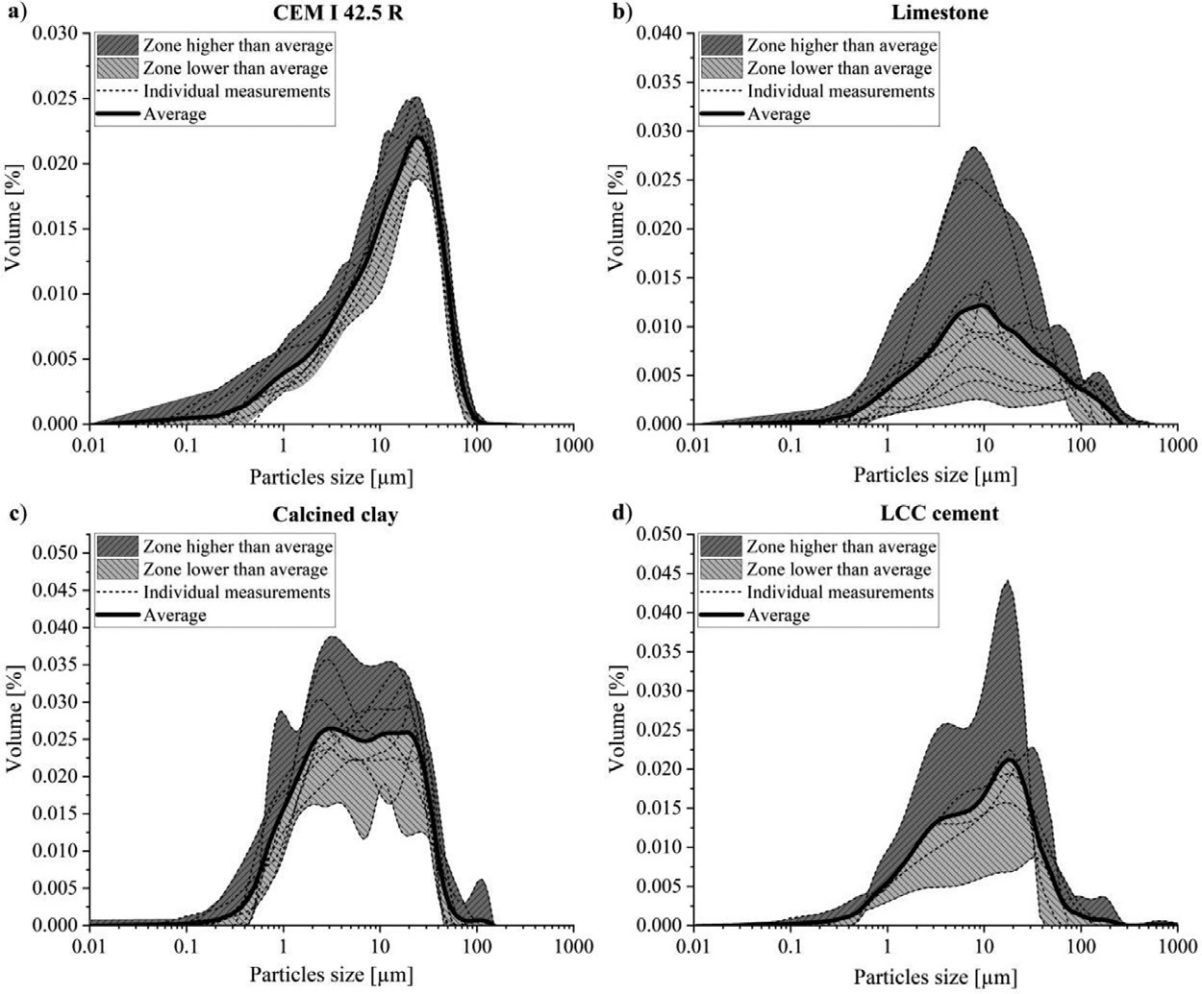
Particle size distributions of (a) CEM I 42.5 R, (b) limestone powder, (c) calcined clay and (d) LCC 70:30 measured by laser diffraction method.

The particle size distributions (PSD) of CEM I 42.5 R, limestone powder, calcined clay and LCC 70:30 were measured by the laser diffraction method according to ISO 13,320: 2020 [10]. The curves were normalized by setting the area below each graph equal to one. The results are presented in Fig. 7. The shadow areas below and above the average line indicate the scope of the testing results, i.e. highlight potential differences among the participating laboratories. The dashed lines show the individual measurements. Results of CEM I 42.5 R and limestone are based on nine, LCC 70:30 of seven and calcined clay of six measurements. The reproducibility of the PSD measurement of the CEM I 42.5 R is very good. The other materials show much lower reproducibility, particularly the limestone powder. To check whether the large fluctuations were due to different subbatches, a separate round robin test was performed. However, the results also showed large variations as well.

**Fig. 8:**
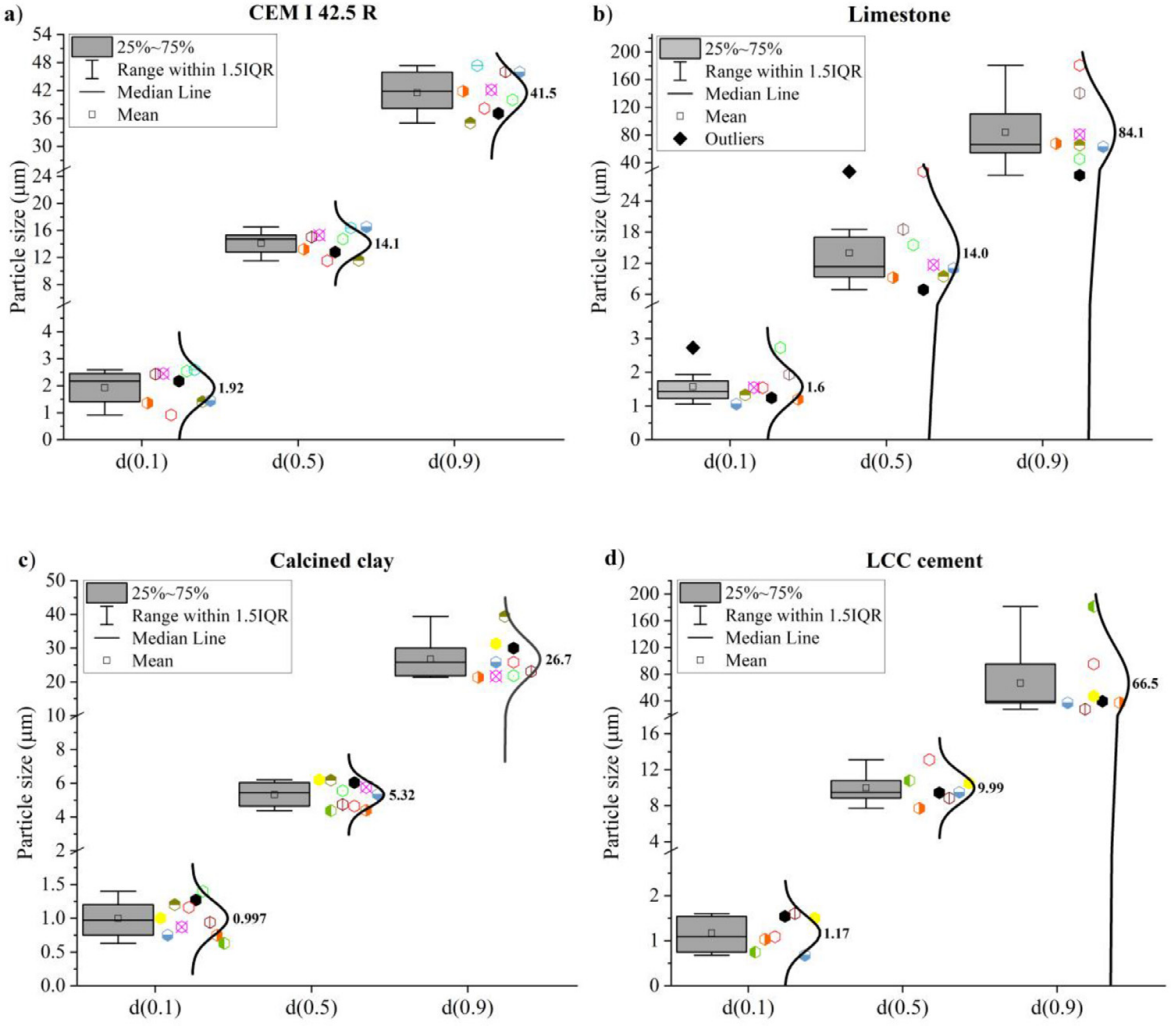
D-values [d(0.1), d(0.5) and d(0.9)] of (a) CEM I 42.5 R, (b) limestone powder, (c) calcined clay and (d) LCC 70:30.

The characteristic d-values d(0.1), d(0.5), and d(0.9) are shown in Fig. 8.

### 2.3 Characterization Data of Further Properties

**Fig. 9:**
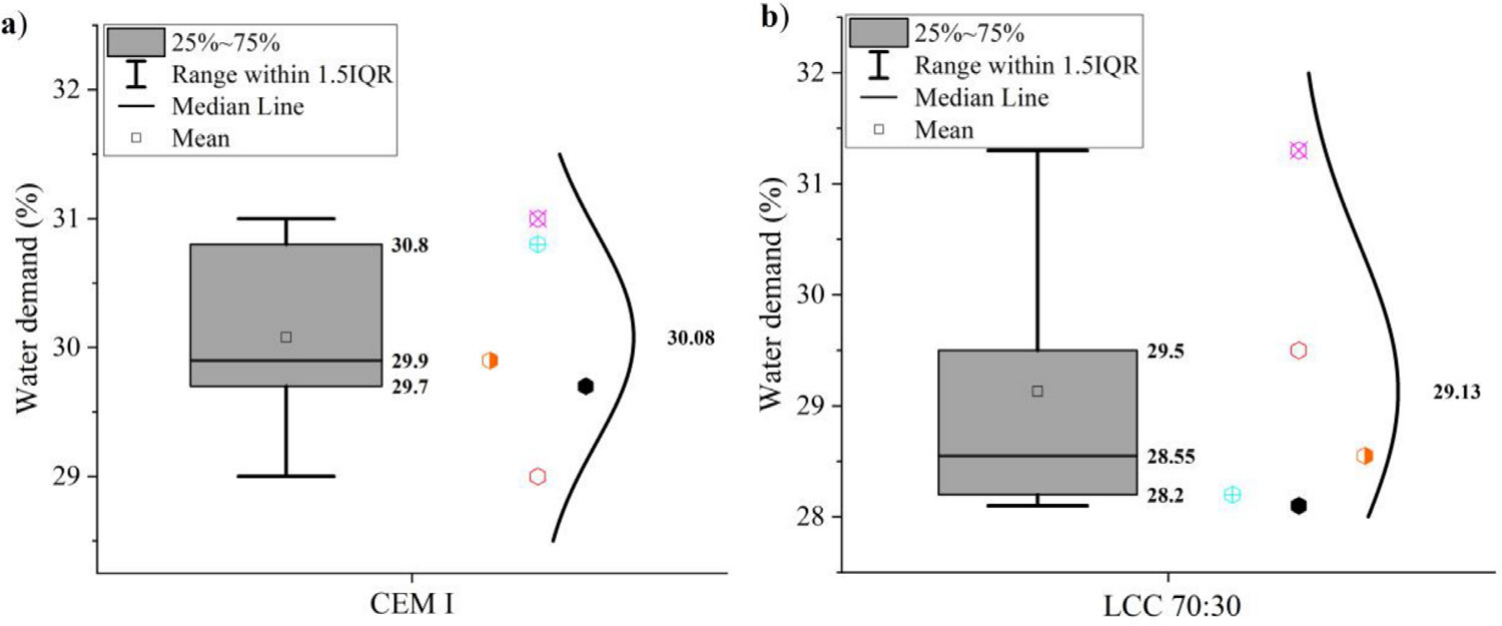
Water demand of (a) CEM I 42.5 R and (b) LCC 70:30.

**Fig. 10:**
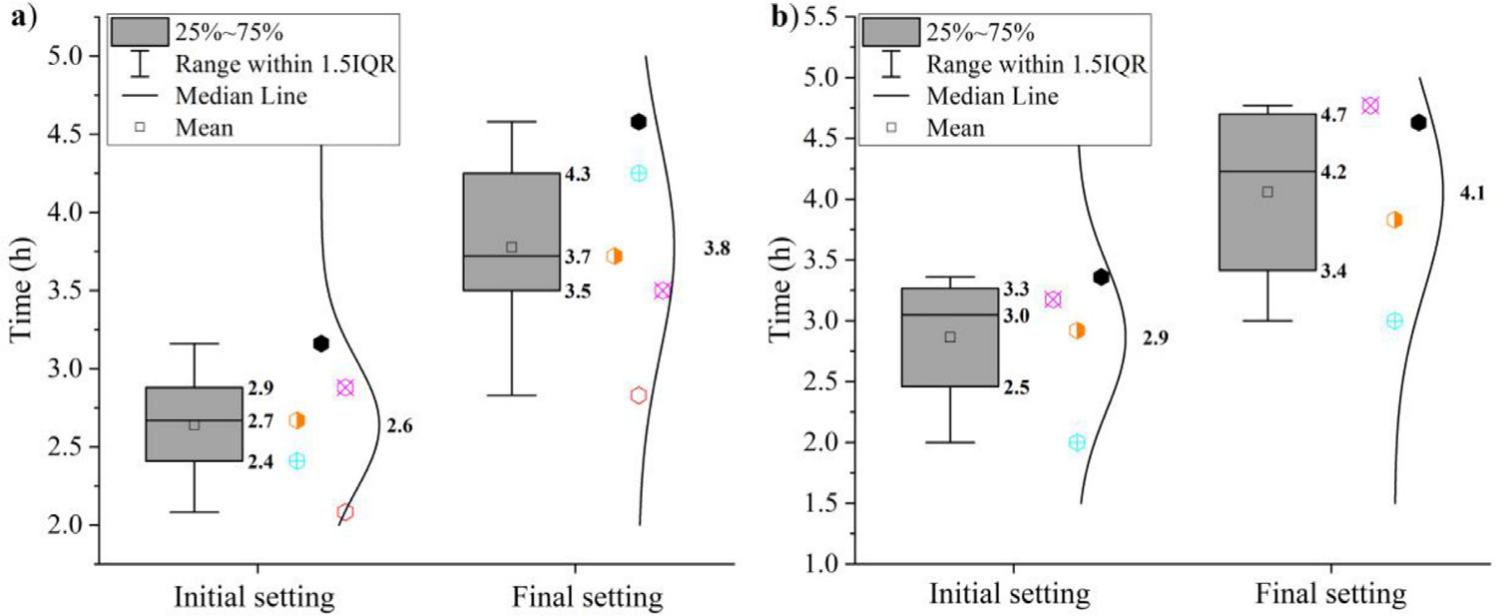
Initial and final setting times of (a) CEM I 42.5 R and (b) LCC 70:30.

**Fig. 11:**
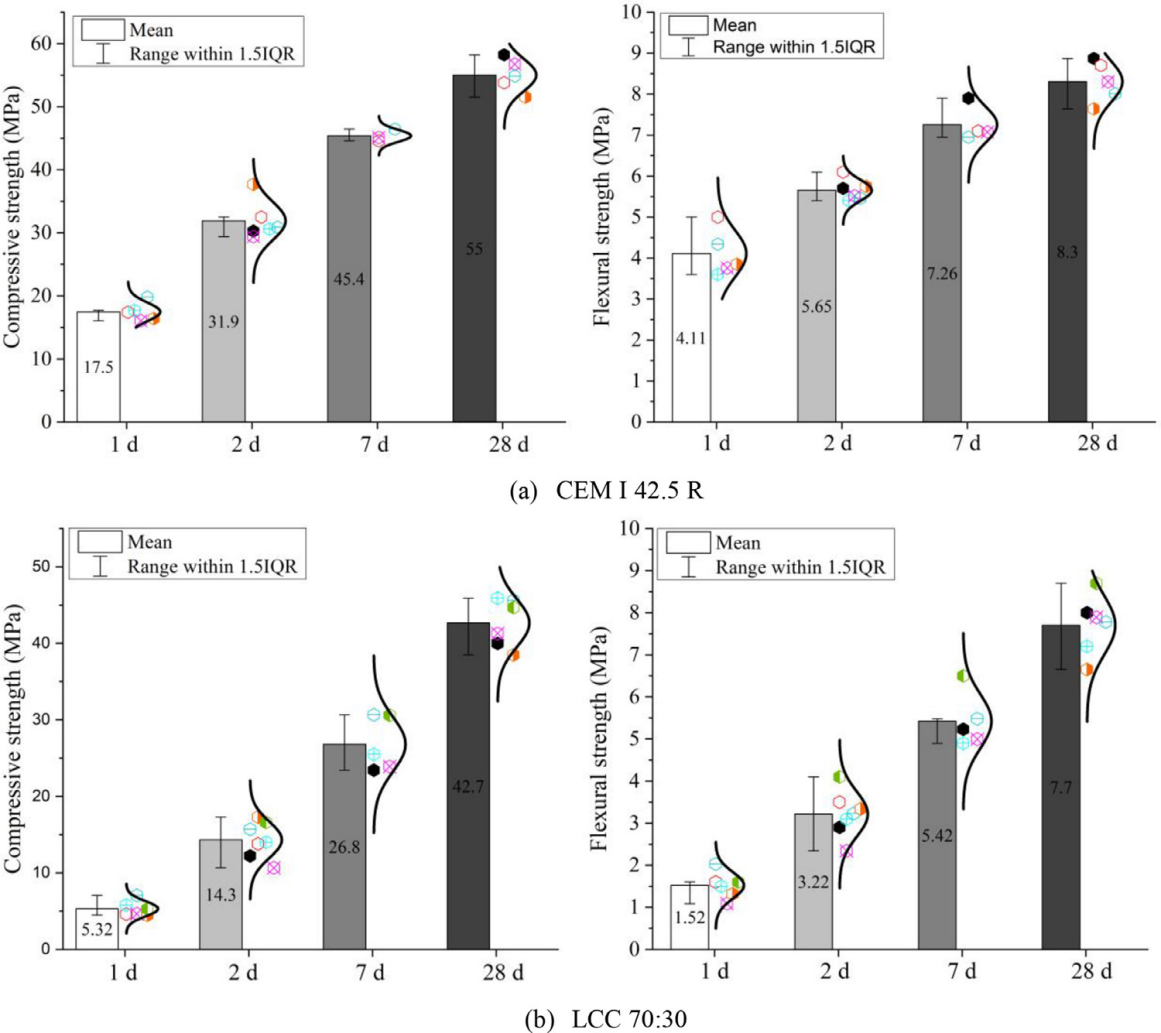
Compressive and flexural strengths of (a) CEM I 42.5 R and (b) LCC 70:30 after 1d, 2d, 7d and 28d.

**Fig. 12:**
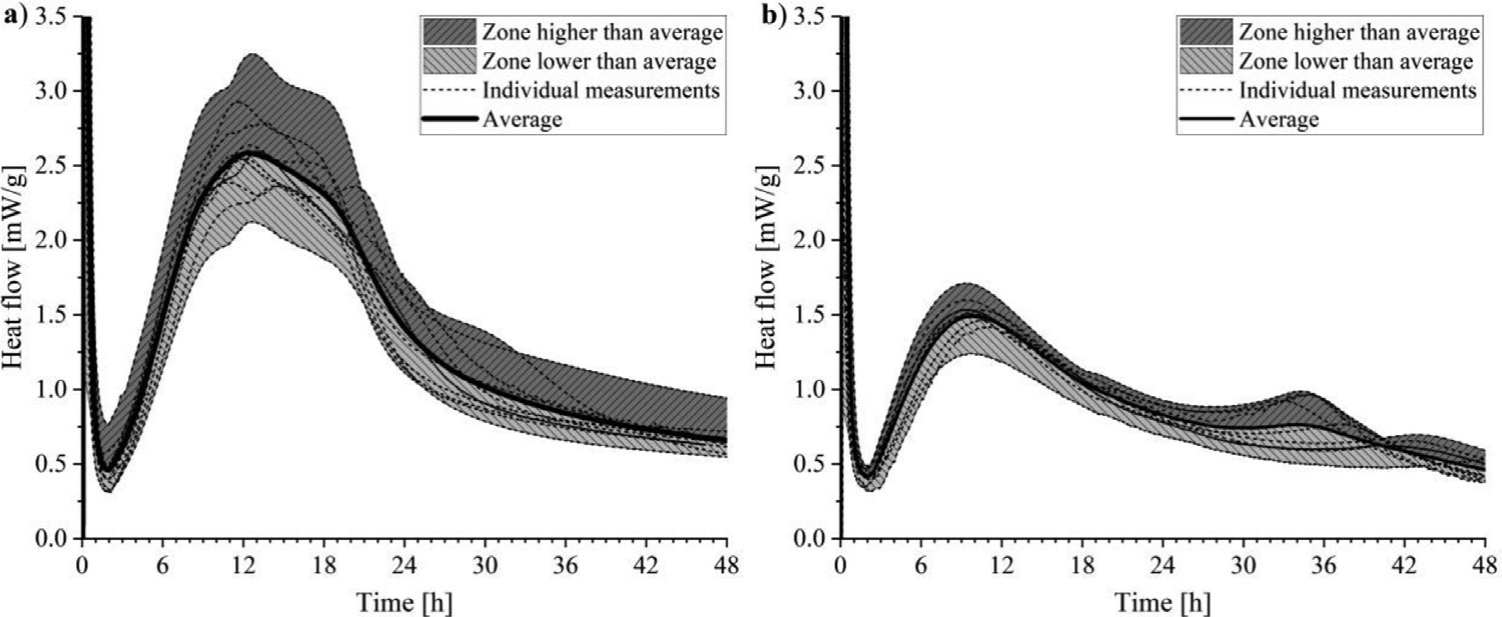
Heat flow curves measured by isothermal calorimetry of (a) CEMI 42.5 R and (b) LCC 70:30 with water to cement ratio of 0.434 at a temperature of 20 °C.

Some additional properties of the samples CEM I 42.5 R and LCC 70:30 are shown in Fig. 9, Fig. 10, Fig. 11, Fig. 12. The water demand can be seen in Fig. 9. Fig. 10 shows the initial and final setting times. Both tests were measured according to EN 196–3: 2017 [11].

Flexural and compressive strengths are shown in Fig. 11. These tests were performed according to EN 196–1: 2016 [12].

The results of isothermal heat flow calorimetry are presented in Fig. 12. The tests were performed according to the method described in EN 196–11: 2019 [13] with a water to cement ratio of 0.434 at a temperature of 20 °C. The shadow areas below and above each average line indicate the scope of the test results. The dashed lines show the individual measurements.

## 3 Experimental Design, Materials and Methods

The material of all groups originates from the same batch and was stored in closed containers. Most of the tests were performed according to the strict procedures described in the following standards. EN 196–2: 2013 was used for oxide composition, insoluble residue and loss on ignition, EN 1097–7: 2008 for the true density, EN 196–6: 2018 for the specific surface area by the Blaine method, ISO 9277: 2010 for the specific surface area by the BET method, EN 196–3: 2016 for water demand and setting times, EN 196–1: 2016 for flexural and compressive strengths, EN 196–11: 2018 for isothermal heat flow calorimetry and ISO 13,320: 2009 for particle size distribution. Tests that are not part of a standard are described in more detail below.

Scanning Electron Microscope (SEM) images were recorded on uncoated powders with a Zeiss GeminiSEM 500 NanoVP, Jena, Germany. A backscatter detector (BSD1) in a low vacuum was used for image acquisition. The electrons were accelerated in an electric field with a voltage of 15 kV. The images were taken with magnifications of 1000, 2000 or 5000, respectively.

For the characterization of phase contents, powder-XRD combined with quantification of the patterns was used. In different research groups, different XRD instruments with different analysis software were used as shown in Table 2.

**Table 2:** Information about the instruments for the XRD measurements and quantification method.

	Affiliation no.				
	2	3	6	9	10
Instrument	Bruker D8 Advance	Bruker D8	Empyrean	Bruker D2	Seifert 3003 TT
Software	Topas - Rietveld Method	Topas - Rietveld Method	Highscore plus 4.8 - Rietveld Method	Topas - Rietveld Method	Autoquan - Rietveld Method
Quantification method	External standard quantification	External standard quantification	Internal standard quantification	Internal standard quantification	Internal standard quantification

## Ethics Statement

This work did not involve human subjects, animal experiments and data collected from social media platforms

## CRediT authorship contribution statement

**U. Pott:** Conceptualization, Formal analysis, Investigation, Writing – original draft, Writing – review & editing, Visualization. **C. Crasselt:** Investigation, Writing – review & editing. **N. Fobbe:** Investigation, Writing – review & editing. **M. Haist:** Resources, Supervision, Funding acquisition. **M. Heinemann:** Investigation. **S. Hellmann:** Investigation. **D. Ivanov:** Investigation, Writing – review & editing. **C. Jakob:** Investigation. **D. Jansen:** Resources, Supervision, Funding acquisition. **L. Lei:** Investigation, Supervision. **R. Li:** Investigation, Writing – review & editing. **J. Link:** Investigation, Writing – review & editing. **D. Lowke:** Resources, Supervision, Funding acquisition. **V. Mechtcherine:** Resources, Supervision, Funding acquisition. **J. Neubauer:** Resources, Supervision, Funding acquisition. **D. Nicia:** Investigation, Writing – review & editing. **J. Plank:** Resources, Supervision, Funding acquisition, Writing – review & editing. **S. Reißig:** Investigation. **T. Schäfer:** Resources, Supervision, Funding acquisition. **C. Schilde:** Resources, Supervision, Funding acquisition. **W. Schmidt:** Resources, Supervision, Funding acquisition. **C. Schröfl:** Investigation, Writing – review & editing. **T. Sowoidnich:** Resources, Supervision, Funding acquisition. **B. Strybny:** Investigation. **N. Ukrainczyk:** Investigation. **J. Wolf:** Investigation. **P. Xiao:** Investigation, Writing – review & editing. **D. Stephan:** Conceptualization, Resources, Writing – original draft, Writing – review & editing, Supervision, Funding acquisition.

## Declaration of Competing Interest

The authors declare that they have no known competing financial interests or personal relationships that could have appeared to influence the work reported in this paper.

## Figures and Tables

**Table A.1.1:** Mean and standard deviation (SD) of the oxide composition of CEM I 42.5 R.

	Composition [wt.-%]															
	CaO	SiO_2_	Al_2_O_3_	Fe_2_O_3_	MgO	K_2_O	Na_2_O	TiO_2_	P_2_O_5_	Mn_2_O_3_	SO_3_*	(SO_3_#)	LOI	Cl^−^	Sum	Insoluble residue
Mean	61.79	21.14	5.53	2.27	1.39	0.77	0.21	0.32	0.14	0.03	2.84	(2.98)	2.48	0.030	98.94	0.82
SD	2.17	1.34	0.42	0.08	0.22	0.08	0.08	0.06	0.08	0.03	0.69	(0.36)	0.10	0.007	–	0.30

⁎ Measured by XRF; included in the sum.

# Analysis by other methods; not included in the sum.

**Table A.1.2:** Mean and standard deviation (SD) of the phase content of CEM I 42.5 R.

	Composition [wt.-%]									
	C_3_S	C_2_S	C_3_A (orth.)	C_3_A (cub.)	C_4_AF	Sulfate carrier	Calcite	Quartz	Periclase	Sum
Mean	54.45	18.07	3.28	7.55	5.17	4.82	4.08	0.42	0.75	98.59
SD	4.15	0.34	1.10	0.89	2.66	1.12	1.87	0.17	0.21	–

**Table A.1.3:** Mean and standard deviation (SD) of the physical properties of CEM I 42.5 R.

				Particle size [μm]		

	Density [g/cm^3^]	Specific surface area*[cm^2^/g]	Specific surface area #[m^2^/g]	d(0.1)	d(0.5)	d(0.9)
Mean	3.139	3499.4	1.038	1.92	14.10	41.49
SD	0.027	149.8	0.111	0.64	1.91	4.33

⁎ Measured by Blaine method.

# Measured by BET method.

**Table A.1.4:** Mean and standard deviation (SD) of other properties of CEM I 42.5 R.

	Water demand [wt.-%]	Setting time [h]		Compressive strength [MPa]				Flexural strength [MPa]			
		Initial	Final	1 d	2 d	7 d	28 d	1 d	2 d	7 d	28 d
Mean	30.08	2.64	3.78	17.47	31.89	45.40	55.03	4.11	5.65	7.26	8.30
SD	0.82	0.42	0.68	1.47	3.02	0.96	2.60	0.57	0.26	0.43	0.50

**Table A.2.1:** Mean and standard deviation (SD) of the oxide composition of limestone powder.

	Composition [wt.-%]															
	CaO	SiO_2_	Al_2_O_3_	Fe_2_O_3_	MgO	K_2_O	Na_2_O	TiO_2_	P_2_O_5_	Mn_2_O_3_	SO_3_*	(SO_3_#)	LOI	Cl^−^	Sum	Insoluble residue
Mean	52.73	2.45	0.64	0.37	0.36	0.09	0.02	0.05	0.03	0.03	0.04	(0.28)	41.81	0.01	98.63	2.02
SD	2.02	0.37	0.06	0.06	0.10	0.03	0.02	–	0.05	0.03	0.05	(0.39)	0.55	–	–	0.34

⁎ Measured by XRF; included in the sum.

# Analysis by other methods; not included in the sum.

**Table A.2.2:** Mean and standard deviation (SD) of the phase content of limestone powder.

	Composition [wt.-%]				
	Calcite	Quartz	Dolomite	Muscovite	Sum
Mean	95.99	2.14	2.09	0.76	100.98
SD	2.01	0.69	1.86	–	

**Table A.2.3:** Mean and standard deviation (SD) of the physical properties of limestone powder.

				Particle size [μm]		

	Density [g/cm^3^]	Specific surface area* [cm^2^/g]	Specific surface area # [m^2^/g]	d(0.1)	d(0.5)	d(0.9)
Mean	2.74	3973.6	1.81	1.57	13.99	84.11
SD	0.01	166.9	0.11	0.54	7.33	50.84

⁎ Measured by Blaine method.

# Measured by BET method.

**Table A.3.1:** Mean and standard deviation (SD) of the oxide composition of calcined clay.

	Composition [wt.-%]														
	CaO	SiO_2_	Al_2_O_3_	Fe_2_O_3_	MgO	K_2_O	Na_2_O	TiO_2_	P_2_O_5_	Mn_2_O_3_	SO_3_*	(SO_3_#)	LOI	Sum	Insoluble residue
Mean	5.25	52.57	21.69	8.12	2.35	3.02	0.33	1.04	0.27	0.09	0.78	(1.21)	3.84	99.35	58.96
SD	0.53	2.09	0.41	0.78	0.43	0.28	0.08	0.08	0.06	0.10	0.36	(0.41)	0.69		1.61

⁎ Measured by XRF; included in the sum.

# Analysis by other methods; not included in the sum.

**Table A.3.2:** Mean and standard deviation (SD) of the phase content of calcined clay.

	Composition [wt.-%]									
	Hematite	Phengite	Feldspar	White Mica	Anorthite	Gypsum	Calcite	Quartz	Gehlenite	Sum
Mean	1.19	3.9	4.80	12.7	6.10	2.7	2.05	19.54	1.9	54.88
SD	0.37	–	3.11	–	2.65	0.6	2.70	2.73	–	

**Table A.3.3:** Mean and standard deviation (SD) of the physical properties of calcined clay.

				Particle size [μm]		

	Density [g/cm^3^]	Specific surface area* [cm^2^/g]	Specific surface area # [m^2^/g]	d(0.1)	d(0.5)	d(0.9)
Mean	2.62	12,929	6.26	1.00	5.32	26.71
SD	0.03	1580	0.56	0.25	0.73	5.97

⁎ Measured by Blaine method.

# Measured by BET method.

**Table A.4.1:** Mean and standard deviation (SD) of the oxide composition of LCC 70:30.

	Composition [wt.-%]														
	CaO	SiO_2_	Al_2_O_3_	Fe_2_O_3_	MgO	K_2_O	Na_2_O	TiO_2_	P_2_O_5_	Mn_2_O_3_	SO_3_*	(SO_3_#)	LOI	Cl^−^	Sum
Mean	42.53	28.12	9.43	3.47	1.43	1.31	0.18	0.43	0.13	0.09	3.25	(2.98)	9.15	0.046	99.57
SD	1.88	2.16	0.67	0.27	0.23	0.10	0.01	0.05	0.03	0.01	0.60	(0.20)	0.30	–	

⁎ Measured by XRF; included in the sum.

# Analysis by other methods; not included in the sum.

**Table A.4.2:** Mean and standard deviation (SD) of the physical properties of LCC 70:30.

				Particle size [μm]		

	Density [g/cm^3^]	Specific surface area* [cm^2^/g]	Specific surface area # [m^2^/g]	d(0.1)	d(0.5)	d(0.9)
Mean	2.89	6528.3	2.65	1.17	9.99	66.52
SD	0.02	560.5	0.15	0.38	1.72	55.24

⁎ Measured by Blaine method.

# Measured by BET method.

**Table A.4.3:** Mean and standard deviation (SD) of other properties of LCC 70:30.

		Setting time [h]		Compressive strength [MPa]				Flexural strength [MPa]			

	Water demand [wt.-%]	Initial	Final	1 d	2 d	7 d	28 d	1 d	2 d	7 d	28 d
Mean	29.13	2.86	4.06	5.32	14.33	26.81	42.66	1.52	3.22	5.42	7.70
SD	1.33	0.60	0.82	1.00	2.39	3.56	3.15	0.32	0.54	0.64	0.70

## Data Availability

Characterization data of reference materials used for phase II of the priority program DFG SPP 3 2005 “Opus Fluidum Futurum – Rheology of reactive, multiscale, multiphase construction materials” (Original data) (DepositOnce).

## Appendix: Average values and the standard deviation calculated based on the results from different groups

A.1 CEM I 42.5 R Table A.1.1, Table A.1.2, Table A.1.3, Table A.1.4.

A.2 Limestone Table A.2.1, Table A.2.2, Table A.2.3.

A.3 Calcined clay Table A.3.1, Table A.3.2, Table A.3.3.

A.4 LCC 70:30 Table A.4.1, Table A.4.2, Table A.4.3.
